# Rationale, design and critical end points for the Riluzole in Acute Spinal Cord Injury Study (RISCIS): a randomized, double-blinded, placebo-controlled parallel multi-center trial

**DOI:** 10.1038/sc.2015.95

**Published:** 2015-06-23

**Authors:** M G Fehlings, H Nakashima, N Nagoshi, D S L Chow, R G Grossman, B Kopjar

**Affiliations:** 1 Division of Neurosurgery and Spinal Program, Department of Surgery, University of Toronto, Toronto Western Hospital, Toronto, Ontario, Canada; 2 Department of Orthopedic Surgery, Nagoya University Graduate School of Medicine, Nagoya, Japan; 3 Department of Orthopaedic Surgery, Keio University School of Medicine, Tokyo, Japan; 4 Department of Pharmacological and Pharmaceutical Sciences, University of Houston, Houston, TX, USA; 5 Department of Neurosurgery, Houston Methodist Hospital,Houston,TX,USA; 6 Department of Health Services, University of Washington, Seattle, WA, USA

## Abstract

**Background:**

Riluzole is a sodium channel-blocking agent used in treating amyotrophic lateral sclerosis. It has been approved by the U.S. Food and Drug Administration, Canadian and Australian authorities, and in many other countries. A phase I trial of riluzole for acute spinal cord injury (SCI) provided safety and pharmacokinetic data and suggested neuroprotective benefits. A phase IIB/III double-blinded randomized controlled trial (RCT) started in January 2014 (https://clinicaltrials.gov, NCT01597518). This article describes the pathophysiological rationale, preclinical experience and design of the phase IIB/III RCT of Riluzole in Acute Spinal Cord Injury Study (RISCIS).

**Objectives:**

The primary objective of the trial is to evaluate the superiority of riluzole, at a dose of 100 mg BID in the first 24 h followed by 50 mg BID for the following 13 days post injury, compared with placebo in improving neurological motor outcomes in patients with C4–C8 level, International Standards for Neurological Classification of Spinal Cord Injury Examination (ISNCSCI) grade A, B or C acute (within 12 h post injury) SCI.

**Setting:**

Acute trauma centers worldwide

**Methods:**

A double-blind, multi-center, placebo-controlled RCT will enroll 351 participants randomized 1:1 to riluzole and placebo. The primary end point is the change between 180 days and baseline in ISNCSCI Motor Score. This study has 90% power to detect a change of nine points in ISNCSCI Motor Score at one-sided *α*=0.025.

**Results:**

Currently enrolling in 11 centers.

**Conclusion:**

This study will provide class I evidence regarding the safety and neuroprotective efficacy of riluzole in patients with acute cervical SCI.

## Introduction

### Background and rationale

Spinal cord injury (SCI) is a devastating event resulting in severe neurological deficit, loss of function and deterioration in quality of life. The annual incidence is 15–40 cases per million, and there are more than one million people living with SCI in North America.^1^ The annual cost of SCI in North America exceeds seven billion dollars,^1^ and the impact is immense at a personal, family and societal level.

During the last decade, a number of therapies have been investigated in clinical trials bringing new hope to patients with SCI.^2^ However, effective therapies, shown to improve neurological and functional recovery, remain absent.

Riluzole is a benzothiazole anticonvulsant drug that is approved for use in amyotrophic lateral sclerosis (ALS) by the U.S. Food and Drug Administration (FDA) and by the regulatory authorities in numerous other countries and jurisdictions.^3^ Riluzole modulates excitatory neurotransmission, and the neuroprotective mechanisms have been shown to improve survival in the setting of ALS.^3^ Preclinical studies of riluzole in the setting of SCI have also demonstrated functional recovery by preventing the aberrant release of sodium and glutamate imbalance.^4, 5^ As such, riluzole is an appealing agent for translation into clinical trials for SCI because of its well-defined human safety record over the past two decades in the treatment of ALS.

A phase I clinical trial investigating the safety and pharmacokinetics of Riluzole in acute SCI was completed in 2011 (https://clinicaltrials.gov no. NCT00876889),^6^ and motor scores were seen to improve for riluzole-treated cervical injury patients on the International Standards for Neurological Classification of Spinal Cord Injury Examination (ISNCSCI), compared with a nonconcurrent comparison group treated with standard of care. A phase IIB/III randomized multi-center controlled trial evaluating the efficacy and safety of riluzole in the management of patients with acute SCI entitled the Riluzole in Acute Spinal Cord Injury Study (RISCIS) commenced in January 2014 (https://clinicaltrials.gov, registration number NCT01597518). The completion of the RISCIS study will provide level 1 evidence either confirming or refuting efficacy of riluzole in the treatment of acute SCI.

#### Pathobiology of SCI

**Figure 1:**
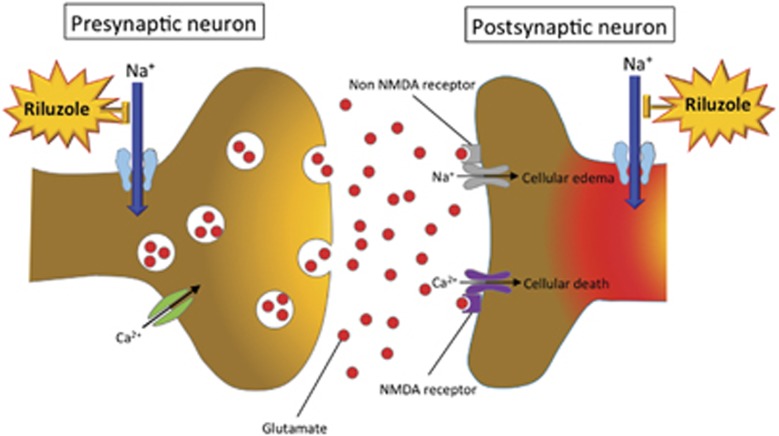
Schematic image of the primary mechanism by which riluzole attenuates the secondary injury in SCI. During the early stage of secondary injury, neuronal ionic balance is disrupted and the intracellular sodium concentration increases as a result of trauma-induced activation of voltage-sensitive sodium channels. The increase in intracellular sodium concentration also promotes concomitant influx of calcium ions, resulting in the development of intracellular acidosis. The excessive influx of sodium and calcium triggers pathologic extracellular release of excitatory neurotransmitter glutamate, leading to cytosolic edema and cellular death. Riluzole blocks the sodium channels in neurons and prevents the increase in intracellular sodium concentration, contributing to the inhibition of cellular death.

The pathobiology of acute SCI involves a primary mechanical injury followed by the secondary injury resulting in further damage. The primary injury involves an array of complex biomechanical forces including acute contusion, compression or laceration due to displacement of bone or disc, and shear stresses loaded on axons or blood vessels. This primary event initiates a post-lesion signaling cascade of downstream events, known as the secondary injury. Petechial hemorrhage in the gray matter and edema in the white matter occur, and thrombosis and vasospasm in microvasculature lead to ischemia of neuronal tissues.^7^ The ischemia leads to neuronal membrane dysfunction, which includes the abnormal continuous activation of neuronal voltage-dependent sodium channels (Figure 1).^7^ This activation causes an increase in intracellular sodium levels. The combination of events following the influx of sodium ions leads to regional cell death, and is the main pathogenesis of secondary neural injury. This mechanism of secondary injury provides the rationale for the use of a sodium channel-blocking agent to reduce the extent of injury.

An intervention to mitigate damage caused by the primary injury in SCI is unlikely; however, the opportunity to preserve remaining viable neurological tissue by mitigating the evolution of secondary injury could result in improved post-injury outcomes.

#### Treatments for SCI

Clinical guidelines for the management of SCI emphasize the need for decompression of the spinal cord, restoration of spinal stability and cardiopulmonary and metabolic support. Currently, there are few therapeutic treatments demonstrating functional outcome improvement in human SCI. Clinical trials with methylprednisolone (NASCIS II and III)^8^ and GM-1 ganglioside^9^ have been performed without strong positive results. A recent prospective, multi-center study suggests that early decompression within the first 24 h post injury is associated with better neurological outcomes than later surgery.^10^

#### Evidence for use of riluzole in SCI

Riluzole is a sodium channel-blocking benzothiazole anticonvulsant.^3^ SCI results in a deleterious accumulation of intracellular sodium level ([Na+]i) through voltage-gated Na+ channels within neural axons,^11^ and dysfunction of membrane-bound Na+–K+-ATPase pump with a reduction in Na+ efflux.^12^ The resulting membrane depolarization associated with cellular inability to remove [Na+]i favors further Na+ influx via the Na+ channels. The marked increase [Na+]i leads to an influx of Ca2+ through Na+–Ca2+ exchange pump. This Ca2+overload stimulates a variety of Ca2+-dependent enzyme systems such as calpains and phospholipases, leading to structural and functional injury.^13^ The neuroprotective effects of riluzole appear to result from a blockade of sodium channels, and prevention of exaggerated Ca2+ influx (Figure 1).^14^ In addition, riluzole has a role as an anti-glutamatergic agent via the inhibition of glutamate release, the prevention of glutamate receptor hypofunction and the increase of glutamate uptake by activating glutamate transporters.^15, 16^ The multifaceted effects of riluzole on excitotoxicity and neuromodulation make it a promising neuroprotective treatment option after SCI. Dr Fehlings' group confirmed the effect of riluzole in SCI using a cervical injury model in rats by comparing it with other sodium channel blockers.^4^ Functional neurological recovery was achieved only with riluzole, and significant long-term tissue sparing and a reduction of cavity area were observed.

#### Optimal timing for administration of riluzole in SCI

Extracellular glutamate rises to a toxic level within 15 min after SCI in rats.^4^ Dr Fehlings' group evaluated the timing of riluzole administration in rodents with severe cervical SCI, and demonstrated that treatment initiated at 1 and 3 h post injury contributed to (1) sensory-motor function improvement, (2) improved axonal conduction and (3) reduced apoptosis and inflammation without increased neuropathic pain.^5^ Extrapolating from these results, we estimated a therapeutic time window of 12 h post injury for riluzole in humans, given that the pathobiological changes in SCI peak approximately four times more rapidly in rats than they do in humans.^5^

#### Phase I clinical trial of riluzole in SCI

The phase I clinical trial was completed in 2011 (https://clinicaltrials.gov no. NCT00876889). Thirty-six patients (28 cervical and 8 thoracic) were enrolled at six clinical centers of the North American Clinical Trials Network (NACTN).^6^ The patients enrolled were admitted within 12 h of SCI, and assessed using ISNCSCI as grade A, B or C at admission. Riluzole (50 mg) was administered every 12 h orally or by nasogastric tube, starting within 12 h of injury for 28 doses. A nonconcurrent comparison group was formed of 36 SCI patients who had received standard of care treatment without riluzole. There were no serious adverse effects or death. Increase in liver enzyme and bilirubin levels were found in 14–70% of patients, but these elevations returned to normal levels without serious events. With regard to other medical complications, the specific types of severe and moderate complications such as infection, pulmonary failure or hematological disease, occurred in both groups of patients, with no significant differences in occurrence rates between groups.

Significant ISNCSCI motor score improvement from admission to 90 days in cervical injury patients was observed in the riluzole-treated group. ISNCSCI grade B patients with cervical injury showed the greatest gains in this motor score. In patients with thoracic SCI, significant motor recovery was not observed because patient numbers were small and all had complete paralysis. In general, the ISNCSCI motor scores are not sensitive to segmental clinical recovery in the thoracic region. On the basis of these results, the phase IIB/III clinical trial for cervical acute SCI began in January 2014, and is known as RISCIS.

#### Clinical pharmacokinetics of riluzole in patients with SCI

To obtain information about the pharmacokinetics (PK) and pharmacodynamics (PD) of riluzole and relate that information to toxicity and efficacy outcomes, individual and population pharmacokinetics of enterally administered riluzole were characterized in a Phase I clinical trial.^17^ The peak concentration and 12-h area under the plasma concentration curve (AUC)_(0–12h)_ achieved in SCI patients were lower than those in ALS patients on the same dose basis, owing to a higher clearance and larger volume of distribution in SCI patients. The finding in SCI patients of large interpatient variability in plasma concentration and an increase in the clearance and distribution of riluzole between the 3rd and 14th days after SCI, with a lower plasma concentration of riluzole on the 14th day, stressed the importance of monitoring changes in drug metabolism after SCI in interpreting the safety and efficacy of therapeutic drugs that are used in clinical trials in SCI.

### Objectives

The primary objective of the RISCIS study is to compare neurologic motor recovery at 6-month follow-up between adult patients with acute SCI receiving either riluzole or a placebo for the same duration after acute SCI. As secondary objectives, the impact of this riluzole regimen on sensory recovery, functional outcomes, quality of life outcomes, health utilities, as well as on mortality and adverse event rates will be evaluated. The study hypothesis is that subjects with acute SCI treated with riluzole will experience superior neurological, functional and quality of life outcomes, as assessed using established measures, at follow-up points to 12 months as compared with those receiving placebo.

### Trial design

RISCIS is a randomized, double-blinded, multi-center, placebo-controlled, two-arm parallel group superiority trial with a sequential adaptive design.. This trial has been registered with https://clinicaltrials.gov (no. NCT01597518). The trial follows applicable institutional and governmental regulations concerning the involvement of human subjects in clinical research. The study sponsor is AO North America Charitable Foundation and AOSpine North America (Chi Lam Project Manager, AOSpine North America, Clam@aospine.org), a nonprofit foundation for excellence in spine.

## Materials and Methods

### Study setting

The investigational sites are selected from the AOSpine North America Research Network, a clinical research consortium funded by AOSpine North America, and the North American Clinical Trials Network (NACTN) for Treatment of Spinal Cord Injury sponsored by the Christopher Reeve Foundation and supported by the U. S. Department of Defense. It is planned, pending funding, that additional sites worldwide will join the study. The central trial management center is at the AOSpine Methods Core where the central electronic online data capture system is held. Dr Michael G Fehlings is the Principal Investigator and chairs the trial Steering Committee and Dr Robert G Grossman is the Co-Principal Investigator. The trial Steering Committee also consists of several content experts, a pharmacologist and a statistician. The consortium centers are listed in Table 1. All treatment sites are primary care research hospitals and clinics. Currently, 14 sites are in the United States, two are in Australia and one is in Canada. At each of these sites, there is a designated primary site investigator supported by at least one professional study coordinator, who is responsible for day-to-day operations. Before commencing enrollment, all sites received research ethics board approval and training in study operations by the AOSpine Methods Core.

### Eligibility criteria

Detailed inclusion and exclusion criteria are provided in Table 2.

#### Main inclusion criteria

1. SCI with ISNCSCI Impairment Scale Grade 'A,' 'B' or 'C' and neurological level of injury between C4 and C8 based upon the first ISNCSCI evaluation after arrival at the hospital.
2. Aged between 18 and 75 years.
3. Able to receive the investigational drug within 12 h of injury.

#### Key exclusion criteria

1. History of prior SCI.
2. Injury arising from penetrating mechanism.
3. Significant concomitant head injury defined by a Glasgow Coma Scale score <14 with a clinically significant abnormality on a head CT.
4. Evidence of hepatic or renal impairment.

#### Enrollment and randomization

**Figure 2:**
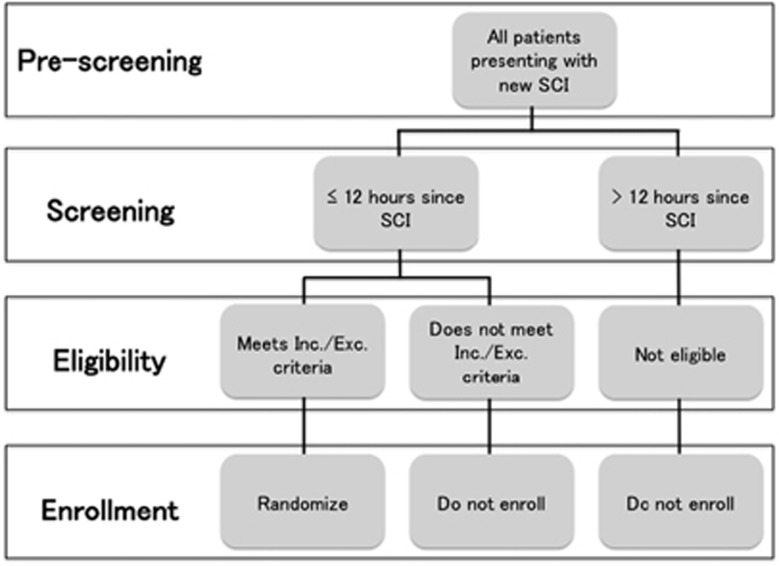
Screening and enrollment design.

Patients who satisfy the inclusion and exclusion criteria (Table 2), agree to study participation and sign the informed consent after being explained all risks and benefits associated with participation in the trial are enrolled and randomized at a ratio of 1:1 to riluzole or placebo arm (Figure 2). The randomization sequence is stratified by site and uses the randomly permuted block sizes of 2 and 4. The randomization sequence is generated by the biostatistician at the central trial management center. For each subject, randomization occurs by opening the lowest sequential number of the sealed randomization envelopes. Each envelope contains a unique number that corresponds to the number on a pre-stocked medication container containing either riluzole or placebo. Throughout randomization and follow-up, the subjects, physicians and data collectors remain blind to treatment allocation. Emergency unblinding procedure for safety reasons is provided.

#### Withdrawal/discontinuation of subjects

A subject will be withdrawn from the study for any of the following reasons:

1. In rare cases, subject may be enrolled before receiving all screening laboratory tests. If the results show clinically significant abnormalities, the subject may be discontinued.
2. Subject voluntarily withdraws consent after enrollment and terminates participation.
3. The investigator withdraws the subject. If this decision is made for safety reasons or noncompliance with the study protocol or procedures, the sponsor/CRO will be notified immediately.
4. The investigator or the sponsor stops the study or stops the patient's participation for medical, safety, regulatory or other reasons consistent with applicable laws, regulations and good clinical practice.

For each case, detailed information will be obtained explaining circumstances leading to the withdrawal. This will be recorded on the Subject Withdrawal Form. Investigational drug assigned to the withdrawn subject shall not be assigned to another subject. The remaining study medication for the withdrawn subject will be obtained from the subject and kept at the site to be processed at the end of the study according to the disposal or return instructions.

For safety reasons, a subject who withdraws from the study for any reason before completion of the dose regimen and the last scheduled lab test will be assessed for safety evaluation purposes. This shall occur within 30 days of the last dose of the investigational drug.

### Interventions: treatment description

Subjects assigned to the active treatment arm receive riluzole at a dose of 100 mg BID in the first 24 h followed by 50 mg BID for the following 13 days after injury. The decision to use the 100-mg loading dose was based on the pharmacokinetics/pharmacodynamics results in the Phase I study. This is an approved FDA dosage and the rationale is to get to optimal therapeutic levels faster. Subjects randomly assigned to the control arm receive a placebo capsule that is identical in shape, size and color to the riluzole capsule for the same duration and at the same interval. The drug is administered by a nurse daily, as it is prescribed and the medications are given to the patient by the nurse according to doctor's orders. There will be, therefore, a medical record of drug administration. External research monitors will perform on-site evaluations to ensure drug adherence (Complete Investigational Drug Log), and make sure that the data are accurate, reliable and complete and that the study was conducted in accordance to the protocol. In addition, there will be monitoring of riluzole plasma levels as in Table 4. At the time of randomization, enrolled subjects receive the medication containers containing the allotted quantity of riluzole or placebo tablets, accompanied with detailed instructions for use. Drug-related compliance is assessed and recorded throughout the study period. Surgical treatment including the approach (anterior or posterior), the type of operation (decompression, fusion or corpectomy) are left to the discretion of the treating surgeon. Postsurgical treatment, including the institution of rehabilitation measures, is left to the standard of care at the participating study site.

### Outcome measures and follow-up

#### Primary efficacy outcome

The primary outcome is change in ISNCSCI total Motor Score (ISNCSCIMS) between baseline and 180 days after injury (Table 3). The ISNCSCI is a universal classification tool for SCI.^18^ The time point of 180 days was chosen based on empirical evidence from an earlier study showing that the majority of functional change and recovery after SCI occurs by this time point.^6^

#### Secondary efficacy outcomes

The trial has two secondary efficacy outcomes.

- Change in ISNCSCI grade between baseline and 180 days
- Spinal Cord Independence Measure (SCIM) III at 180 days

The SCIM^19^ is the only comprehensive rating scale that measures the ability of patients with spinal cord lesions to perform everyday tasks.

#### Other outcomes

Other outcomes consist of health-related quality of life SF-36 version 2, EQ-5D, Pain Numeric Rating Scale (Pain NRS), and sensorimotor upper limb function (Graded Redefined Assessment of Strength Sensibility and Prehension: GRASSP) outcomes (Table 4).^20^

### Safety outcomes

All adverse events are recorded on an ongoing basis throughout the study period. All serious and unexpected adverse events will be reported to the Medical Monitor at the time of occurrence.

### Pharmacological substudy

A subset of clinical centers, specifically nine NACTN centers, are the sites for the pharmacological substudy. It is assumed that a threshold level of blood plasma concentration of riluzole must be reached to achieve a therapeutic effect and that there is a therapeutic range of concentrations. The previously published reports of the pharmacology of riluzole in the Phase I trial reported large differences in maximal concentration of riluzole between patients. It is possible that the low levels of riluzole in some patients did not reach a threshold for efficacy. In grade B patients with cervical injuries, a positive correlation was found between the plasma concentrations and motor outcome scores when extreme peak concentration (*C*_max_) values and motor scores were censored.^6^ The pharmacological substudy aims to determine a safe and clinically effective therapeutic range of plasma concentration of riluzole. If these can be established, monitoring riluzole plasma levels and adjustment of the enteral dose would be a rational approach to therapy.

The specific aims of the pharmacological substudy are to determine the individual peak and trough concentrations of riluzole after enteral administration of the study doses described above. From this, the aim is to derive individual pharmacokinetic parameters of half-life (*t*_1/2_), systemic exposure (AUC_0→24_), volume of distribution (*V*_d_) and clearance by one-compartment model, using Bayesian iterative two-stage procedure. Riluzole concentration will also be correlated with laboratory measures including aspartate aminotransferase, alanine aminotransferase, white blood count and the incidence of adverse events, as well as with neurological outcome scores.

### Participant timeline

Participant timeline is shown in Table 4.

### Sample size, interim analysis and adaptive techniques

The statistical analysis will test the null hypothesis of the superiority of riluzole compared with placebo in change of ISNCSCIMS between the baseline and the 180-day follow-up (Δ ISNCSCIMS).

#### Statistical tests

The statistical testing of *H*_0_ for the primary end point will be organized as a two-stage sequential adaptive design. There will be one interim analysis at about 60% of the accrued sample in addition to the final analysis. The interim analysis has multiple functions:

Testing of *H*_0_ (that is, efficacy)
Testing of H1 (that is, futility, lack of effect)
If none of the above, sample size will be adjusted, if indicated

The overall sequential design will be organized in the following way. The statistical design will address the efficacy and futility. The statistical testing of *H*_0_ hypothesis will be performed as a one-way test with *α* level 0.025, testing the superiority of riluzole arm compared with placebo arm. The superiority of placebo over riluzole (that is, harmful effect of riluzole) will not be tested as it has no clinical implication. *α*-spending for the testing of *H*_0_ will resemble an O'Brien–Fleming distribution. The testing for futility (*H*_1_) will consequently be organized as one-way testing. The *β*-spending for futility testing will follow *γ*-distribution with the parameter (−1). The results of the interim analysis will be reviewed by the DSMB (Data Safety and Monitoring Board) and will not be shared with the sponsor, participating investigators or patients, except in the case that the study reaches termination or withdrawal criteria.

#### Sample size

On the basis of the above statistical design, specifications and empirically derived s.d. for ISNCSCIMS change of 24.08 from a large case series of prospectively followed SCI subjects in an earlier study, a sample size of 316 subjects (158 in each arm) will have 90% power to detect a nine point difference in the Δ ISNCSCIMS at one-sided *α*=0.025. To account for losses to follow-up of up to 10%, the study will enroll 351 subjects.

The sample size estimate is based on certain assumptions. The main assumptions affecting the sample size are that of the true effect size and the s.d. for the difference in the Δ ISNCSCIMS. These assumptions will be verified during the study and sample size adjustment will be performed if needed, using the adaptive techniques. The sample size adjustment will be performed after the first interim analysis of the data.

#### Missing values

Any missing follow-up data will be imputed through a multiple imputation procedure that is less susceptible to bias than the complete case analysis technique. Multiple imputation is the preferred method for handling missing outcome data in therapeutic trials, as recommended by the FDA.

#### Study success

Study will be considered to successfully confirm the working hypothesis if *H*_0_ for the primary end point has been rejected either at interim or the final analysis.

#### Secondary outcomes

Testing for all secondary outcomes will be based on appropriate statistical methods and two-way superiority testing. Secondary outcomes will not be tested at the interim analysis, except if the withdrawn rules were met.

#### Preplanned subgroup analysis

A preplanned subgroup analysis will compare the differences in Δ ISNCSCIMS among the subjects with ISNCSCI Impairment Scale Grade 'A,' 'B' and 'C.'

#### Safety

Safety will be monitored through the course of the study by a designated Safety Officer who is not associated with the Sponsor and is not an investigator in the study. Trends in serious adverse events, laboratory events and unexpected adverse events will be reviewed by external independent DSMB. The DSMB will evaluate safety information against the pre-specified safety stopping rules. The DSMB will also review the results of the interim statistical analysis.

#### Study population

The analysis will be performed on intention-to-treat population.

### Quality assurance

Administration of study medication will be recorded in the Medication Compliance Log. External independent clinical research monitors will perform frequent on-site visits to ensure that the subjects have provided their consent to participate in the study, that the data are true, accurate, reliable and complete, that patient safety is maintained and that all adverse events are evaluated and reported, and that the study is conducted in accordance with the study protocol. Throughout the course of the trial, all subject-related source data will be transcribed into the eCRF online electronic data capture system OpenClinica (OpenClinica, LLC, Waltham, MA, USA), which will be maintained at the central trial management center. Study data in eCRF will be continuously monitored and any inconsistencies resolved through online ticketing system inbuilt into eCRF.

### Publication policy

Trial data are owned by the Study Sponsor. Each investigator will obtain a copy of their site data set. A central data set will be maintained by the AOSpine Methods Core and will be used for all multi-center publications.

### Subject insurance

Sponsor carries subject insurance in case of research-related injury.

### Conclusion and current trial status

Preclinical studies suggest that glutamate-related excitotoxicity contributes to the pathology of SCI. Riluzole, an FDA-approved medication, has been shown to mitigate such excitotoxicity in animal models of traumatic spinal cord injury, leading to improved neurobehavioral outcomes. To investigate the efficacy and safety of riluzole in the treatment of human SCI, a multi-center, double-blinded, randomized controlled trial has been undertaken, and patients are being enrolled currently. At the time of writing, a total of 11 patients have been enrolled in the study.

## Data Archiving

There were no data to deposit.

## Figures and Tables

**Table 1:** Summary of centers participating in the RISCIS Study

*Principal investigator*	*Site*
Nicholas Theodore, MD	Barrow Neurological Institute, Phoenix, AZ, USA
Paul Arnold, MD	Kansas University Medical Center, Kansas City, KS, USA
Ahmad Nassr, MD	Mayo Clinic, Rochester, MN, USA
James Schuster, MD	Hospital of the University of Pennsylvania, Philadelphia, PA, USA
James Harrop, MD	Rothman Institute, Philadelphia, PA, USA
Darrel Brodke, MD	University of Utah, Salt Lake City, UT, USA
Christopher Shaffrey, MD	University of Virginia, Charlottesville, VA, USA
Bizhan Aarabi, MD	University of Maryland, Baltimore, MA, USA
Michele Johnson, MD	University of Texas Health Science Center, Houston, TX, USA
Maxwell Boakye, MD	University of Louisville, Louisville, KY, USA
James Guest, MD	University of Miami, Miami, FL, USA
Joseph Hobbs, MD	Brooke Army Medical Center, Fort Sam Houston, TX, USA
Graham Creasey, MD	Stanford University, Stanford, CA, USA
Ralph Stanford, MD	Prince of Wales Hospital, Sydney, NSW, Australia
Jonathon Ball, MD	Royal North Shore Hospital, Sydney, NSW, Australia
Robert Grossman, MD	Houston Methodist Hospital-NACTN Coordinating Center, Houston, TX, USA
Michael Fehlings, MD, PhD	University of Toronto Spine Program and Toronto Western Hospital, Toronto, ON, Canada

Abbreviations: AZ, Arizona; CA, California; FL, Florida; KS, Kansas; KY, Kentucky; MA, Massachusetts; MN, Minnesota; NSW, New South Wales; ON, Ontario; PA, Pennsylvania; TX, Texas; UT, Utah; VA, Virginia.

**Table 2:** Eligibility inclusion and exclusion criteria

Eligibility inclusion criteria	• Age between 18 and 75 years inclusive • Able to cooperate in the completion of a standardized neurological examination by ISNCSCI standards (includes patients who are on a ventilator) • Willing and able to comply with the study protocol • Informed Consent Document (ICD) signed by patient, legal representative or witness • Able to receive the investigational drug within 12 h of injury • ISNCSCI Impairment Scale Grade 'A,' 'B' or 'C' based upon the first ISNCSCI evaluation after arrival to the hospital • Neurological Level of Injury between C4 and C8 based upon first ISNCSCI evaluation after arrival to the hospital • Women of childbearing potential must have a negative serum β-hCG pregnancy test or a negative urine pregnancy test
Eligibility exclusion criteria	• Injury arising from penetrating mechanism • Significant concomitant head injury defined by a Glasgow Coma Scale score <14 with a clinically significant abnormality on a head CT (head CT required only for patients suspected to have a brain injury at the discretion of the investigator) • Pre-existent neurologic or mental disorder which would preclude accurate evaluation and follow-up (i.e., Alzheimer's disease, Parkinson's disease, unstable psychiatric disorder with hallucinations and/or delusions or schizophrenia) • Prior history of spinal cord injury • Recent history (<1 year) of chemical substance dependency or significant psychosocial disturbance that may impact the outcome or study participation, in the opinion of the investigator • Is a prisoner • Participation in a clinical trial of another Investigational Drug or device within the past 30 days • Hypersensitivity to riluzole or any of its components • Neutropenia measured as ANC measured in cells per microliter of blood of <1500 at screening visit • Creatinine level of >1.2 mg dl^−1^ in males or >1.1 mg dl^−1^ in females at screening visit • Liver enzymes (ALT/SGPT or AST/SGOT) three times the ULN at screening visit • Active liver disease or clinical jaundice • Subject is currently using, and will continue to use for the next 14 days any of the following medications which are classified as CYP1A2 inhibitors or inducers:*
	Inhibitors:
	– Ciprofloxacin – Enoxacin – Fluvoxamine – Methoxsalen – Mexiletine – Oral contraceptives – Phenylpropanolamine – Thiabendazole – Zileuton
	Inducers:
	Montelukast – Phenytoin
	*Note: no washout period required; if these medications are discontinued, subjects are eligible to be enrolled in the trial
	• Acquired immune deficiency syndrome (AIDS) or AIDS-related complex • Active malignancy or history of invasive malignancy within the last 5 years, with the exception of superficial basal cell carcinoma or squamous cell carcinoma of the skin that has been definitely treated. Patients with carcinoma *in situ* of the uterine cervix treated definitely more than 1 year before enrollment may enter the study • Lactating at screening visit

Abbreviations: ALT, alanine transaminase; ANC, absolute neutrophil count; AST, aspartate transaminase; SGOT, serum glutamic oxaloacetic transaminase; SGPT, serum glutamic-pyruvic transaminase.

**Table 3:** Primary and secondary end points

Primary efficacy end point	• Absolute change in the International Standards for Neurological Classification of Spinal Cord Injury Examination (ISNCSCI) Total Motor Score (ISNCSCIMS) between 180 days and baseline
Secondary efficacy end points	• Change in ISNCSCI grade between baseline and 180 days. • Spinal Cord Independence Measure (SCIM) III at 180 days.
Other end points	• Change in ISNCSCI Sensory Scores (Light Touch and Pin Prick) between 180 days and baseline • Change in ISNCSCI Upper Extremity Motor Score between 180 days and baseline • Change in ISNCSCI Lower Extremity Motor Score between 180 days and baseline • Change in Short Form 36 Version 2 (SF-36v2) PCS, MCS and 8 dimensions between 180 days and pre-injury (recall) • Change in EQ-5D health utility between 180 days and pre-injury (recall) • Graded Redefined Assessment of Strength Sensibility and Prehension (GRASSP) at 14 days or Discharge (whichever occurs first) and 180 days • Change in Numeric Pain Rating Scale (pain NRS) at 14 days, 84 days and 180 days

**Table 4:** Schedule of study activities

	*Screening/enrollment*	*Surgery (if applicable)*	*72 ±12 h post injury*	*7±1 day post enrollment*	*14±2 days*	*Discharge from acute care*	*84±14 days*	*180±30 days*	*365±45 days*	*Unscheduled visit*
Sign ICD	×									
Health Information Release Form (if applicable)	×									
Inclusion/exclusion	×									
Obtain demographics	×									
Screening labs, PK plasma	×		×	×	×					
Clinical labs										
Pregnancy test (if applicable)	×		×		×		×	×	×	
ISNCSCI	×									
Randomization	×									
Dispense investigational drug	×									
Complete investigational drug log	×				×					
Medication compliance SW^a^	×				×					
Charlson Comorbidity Score	×									
Injury Severity Score	×									
SF-36v2.0	× b						×	×	×	
EQ-5D	× b						×	×	×	
Obtain and complete socioeconomic and health behavior SWs	×									
Obtain and complete medical history SWs	×									
Spine trauma injury data SW	×									
Concomitant medications	×	×	×	×	×	×	×	×	×	×
Vital signs	×	×	×		×	×				
Record operative data		×								
MRI			× c							
SCIM III					×		×	×	×	
GRASSP					× d	× d		×		
Pain NRS					×		×	×	×	
Report AEs and SAEs (including intraoperative)		×	×	×	×	×	×	×	×	×
Discharge information						×				
Physical and occupational therapy						×	×	×	×	
Verify data and enter into eCRF within 48 h^e^	×	×	×	×	×	×	×	×	×	×

Abbreviations: AE, adverse event; eCRF, electronic case report form; ISNCSCI, International Standards for Neurological Classification of Spinal Cord Injury Examination; MRI, magnetic resonance imaging; NRS, Numeric Rating Scale; SAE, serious adverse event; SCIM, Spinal Cord Independence Measure.

a Medication compliance should be completed daily.

b Recall of status before the injury.

c MRI between 48 and 72 hours at the discretion of the Investigator.

d GRASSP will be performed at 14 days or discharge (which occurs first).

e Data should be entered into the eCRF within 48 h, but no later than 14 calendar days from collection.
